# Serum and supplemental vitamin D levels and insulin resistance in T2DM populations: a meta-analysis and systematic review

**DOI:** 10.1038/s41598-023-39469-9

**Published:** 2023-07-31

**Authors:** Xingxing Lei, Qian Zhou, Yanmei Wang, Shunlian Fu, Zinan Li, Qiu Chen

**Affiliations:** 1 Hospital of Chengdu University of Traditional Chinese Medicine, Chengdu, 610072 Sichuan China; 2 Hospital of Chengdu University of Traditional Chinese Medicine, Sichuan Province, No. 39, Shi-er-Qiao Road, Chengdu, 610072 People’s Republic of China

**Keywords:** Endocrine system and metabolic diseases, Endocrine system and metabolic diseases

## Abstract

Observational studies have shown a negative correlation between Vitamin D level and the likelihood of developing insulin resistance (IR) and/or diabetes over time, yet evidence remains inconsistent. In this meta-analysis and systematic review, we strive to define the potential association between serum or supplemental Vitamin D Levels and insulin resistance respectively, as well as the contribution of Vitamin D to type 2 diabetes, and to summarize the biologic plausibility of Vitamin D. Four databases (PubMed, Embase, Cochrane Library, and Web of Science) were searched for this Systematic Literature Review (SLR) to find appropriate observational studies and clinical trials published in English through to July 2022. EndNote (version X9) is used to manage the literature search results. We calculated Standard Mean Differences (SMDs) and Risk Ratios (RRs) with their 95% Confidence Intervals (CIs), separately, for continuous and dichotomous outcomes. The correlation coefficients were normalized to z values through Fisher’s z-transformation to calculate the relevant statistics. Meta-analyses were carried out for all comparisons, based on a random-effects pooling model. Data analysis was performed using RevMan (version 5.3) and STATA (version 15.1). All statistical tests were two-sided, with P < 0.05 were regarded as significant. In our current meta-analysis, there are 18 RCTs and 20 observational studies including 1243 and 11,063 participants respectively. In the overall analysis, the diabetic with Vitamin D supplement treatment group showed significantly improve serum insulin (SMD =  − 0.265, 95% CI − 0.394 to − 0.136, P < 0.05), glucose (SMD =  − 0.17, 95% CI − 0.301to − 0.039, P < 0.05) and HOMA-IR (SMD =  − 0.441, 95% CI − 0.582 to − 0.3, P < 0.05) compared with the routine treatment group. Correlation analysis results showed that all three outcomes were significantly correlated in a negative manner with raised Vitamin D (insulin: r =  − 0.08 95% =  − 0.12 to − 0.04; glucose: r =  − 0.06 95% =  − 0.11 to − 0.01; HOMA-IR: r =  − 0.08 95% =  − 0.09 to − 0.06). Results of overall analysis proved that vitamin D has shown significant effect on regulates insulin resistance, and there is a significant inverse association between serum Vitamin D level and IR. Vitamin D supplementation is expected to be integrated into conventional medical approaches to prevent type 2 diabetes and to mitigate the burden of diabetes for individuals and society.

PROSPERO registration number: CRD42022348295.

## Introduction

Diabetes mellitus describes a group of metabolic disorders that leads to hyperglycemia resulting from insulin deficiency, whether relative or absolute, as well as peripheral insulin resistance (IR)^1^. The latest estimates of the increasing prevalence of diabetes exceed previous predictions of diabetes in adults demonstrate the significant public health of diabetes and the global prevalence of diabetes has risen progressively over the past 15 years, with significant regional differences^2^. Since the year 2000, The International Diabetes Federation (IDF) has been produced measuring the diabetes prevalence nationally^3^. A recent study by IDF, Diabetes Atlas, found global prevalence of diabetes was estimated to be 10.5% (536.6 million) among individuals aged 20–79 years in 2021, and it is expected to rise to 12.2% (783.2 million) in 2045^4^.

Type 2 Diabetes Mellitus (T2DM) ranging from predominantly relative insulin deficiency in the face of insulin resistance to primarily impaired insulin secretion with or without insulin resistance, which comprises 90–95% of those forms with diabetes^5^. Since T2DM people is characterized with metabolic abnormalities associated with disorders of insulin action and insulin secretion, such as hypertension, elevated Triglycerides and low HDL-cholesterol, which have been identified as risk factors for T2DM^4^. Insulin resistance is the major pathogenic factor of T2DM, which is interrelated and contribute to the development of impaired glucose tolerance, T2DM and complications of diabetes. Despite the choice of pharmacologic agents, identification of therapy measures for T2DM has been challenging because lifestyle and genetic components are interrelated with insulin resistance^6,7^. Moreover, the non-pharmacologic approaches of dietary modification, weight management and exercising regularly are also required stress. Presently, the Diabetes Prevention Program(DPP) is under way to establish which treatment for reducing IR may help initiate prevention measures of T2DM onset^8^.

Although researches showed that macronutrient patterns are predictive of insulin resistance^9^, some micronutrients, particularly vitamin D intake, are closely related to insulin sensitivity^10,11^, as well as T2DM^12^. Specifically, it appears that low of Vitamin D levels seem to be is associated with increased risk of IR. Given VD deficiency can be easily screened, with Vitamin D-fortified supplements being readily and affordably available, the administration of vitamin D to prevent or reduce insulin resistance represents an attractive avenue. As described in the review by Wallace et al.^13^, many studies have assessed the association between Vitamin D status and insulin sensitivity over the past decades. However, the literature indicated that mixed results are commonplace. Until then, Vitamin D is a promising adjuvant therapy, yet unproven dietary intervention for T2DM and reducing risk of diabetes through regulating insulin resistance.

Therefore, we performed the meta-analysis to define the potential association between serum or supplemental Vitamin D Levels and insulin resistance respectively, as well as the contribution of Vitamin D to type 2 diabetes, and to summarize the biologic plausibility of Vitamin D. This literature review attempts to summarize our current evidence on this burgeoning area through a meta-analysis and systematic review of randomized control trials (RCTs) and observational studies, which will help steer the current clinical research directions, guide best practices and indicate future research directions.

## Method

This Systematic Literature Review (SLR) was performed based on the Cochrane Collaboration format and the Preferred Reporting Items for Systematic Reviews and Meta-analysis (PRISMA) guidelines^14^ (Supplementary Table 1) for conducting, reporting and updating of systematic reviews.

### Data sources and search strategy

Four databases (PubMed, Embase, Cochrane Library, and Web of Science) were searched for this SLR to find appropriate observational studies and clinical trials published in English through to July 2022. The reference lists of previous relevant systematic reviews were also reviewed in order to identify additional eligible studies. Unpublished data was searched in the International Standard Randomized Controlled Trial Number Registry and Clinical Trials. The search used both as free keywords and in combination with broadly defined medical subject headings (MeSH) including “Vitamin D (25-hydroxy vitamin D)”, “insulin resistance” and “diabetes” (Supplementary Table 2). The terms of clinical trials literatures included “diabetes” for the Patient; “Vitamin D” were used for Intervention; Routine treatment was used for Comparison and “insulin resistance” was used for mainly Outcome. The search for articles was based on the scientific name of the keywords as well as the common name. EndNote (version X9) is used to manage the literature search results.

### Selection of studies

The randomized clinical trials (RCTs) and observational studies with a control group were eligible. The observational human studies selected that suggested a relation between serum Vitamin D and IR in type 2 diabetes. The clinical trials were regarded as eligible if they (a) were RCTs that evaluated the Efficacy of Vitamin D supplementation on relieving insulin resistance in T2DM patients; and (b) used oral Vitamin D preparation. All observational studies and clinical trials included met the following criteria: (a) studies published in English-language reported at least one of the following primary outcomes: serum Vitamin D concentration, Fasting Blood Glucose (FBG), Homeostasis Model Assessment-Insulin Resistance (HOMA-IR) and Fasting Insulin (FI); and (b) without time period limitations. Exclusion criteria were as follows: (a) other routes of administration excluding oral ingestion; (b) type one diabetes, acute and chronic diabetes complications, other clinically relevant diseases; (c) involving patients aged < 18 years; and (d) review articles, case reports, editorials, poster abstracts and etc.

### Data extraction and quality assessment

Primary information sources were trial reports published in the medical literature and accompanying supplementary materials. The retrieved baseline data were gathered by two researchers separately, included demographic characteristics, interventions and outcome measures. Assessing the literature quality and bias of all eligible RCTs using the Cochrane Collaboration Risk of Bias Tool with seven criteria. Details are as follows: (1) random generation, (2) allocation concealment, (3) blinding of participants and personnel, (4) blinding of outcome, (5) incomplete outcome data, (6) selective reporting and (7) other sources. The risk of bias was classified as unclear, high or low. Grading of Recommendations Assessment, Development, and Evaluation (GRADE) was used to evaluate observational studies according to the gold standard international methodology.

### Data synthesis and analysis

We calculated Standard Mean Differences (SMDs) and Risk Ratios (RRs) with their 95% Confidence Intervals (CIs), separately, for continuous and dichotomous outcomes. Correlation coefficients were normalized to z values via Fisher’s z-transformation to calculate the relevant statistics. Meta-analyses were carried out for all comparisons, based on a random effects pooling model. Cochran’s Q test and I^2^ index and thresholds of I^2^ were used to evaluate the degree of the heterogeneity between studies, which describe the variation due to heterogeneity rather than random error, with P values < 0.25 representing low significant heterogeneity (low), 0.25–0.5 (moderate) and > 0.5 (high). Additional analyses as sensitivity and subgroup analyses were performed to identify the origins of Heterogeneity. Publication bias was retrieved by means of a funnel plot visual inspection and Egger’s test. Data were analyzed using RevMan (version 5.3) and Stata (version 15.1). All statistical tests were two-sided, with P < 0.05 were regarded as significant.

## Results

### Literature screening results

**Figure 1:**
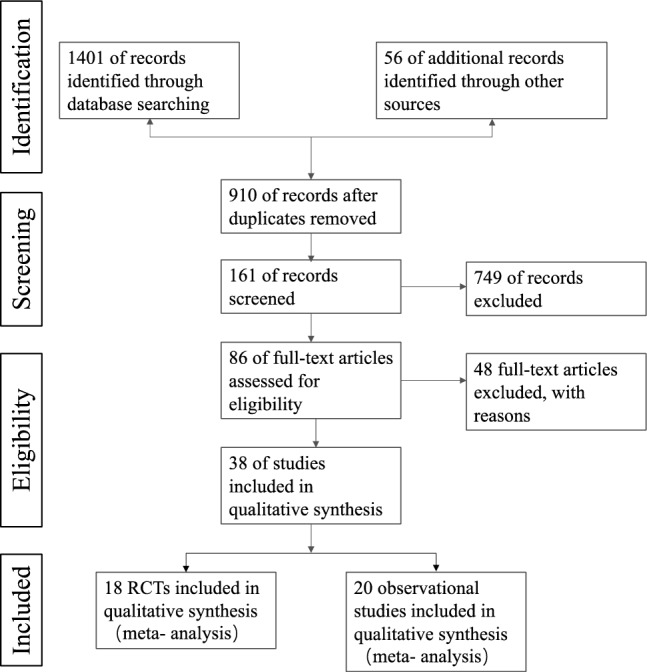
Flowchart of literature search.

A total of 1457 studies were collected electronically from Medline, Embase, PubMed and Cochrane Library and 38 entries were retrieved by hand search. All retrieved results were imported into Endnote X9 software, and 547 duplicate studies were identified deleted. 749 articles were rejected on the basis of title and abstract assessment. After full text evaluation, 18 RCTs and 20 observational studies fulfilled the inclusion criteria and were finally selected as eligible for further meta-analysis. The PRISMA flow diagram of the Systematic Literature Review is presented in Fig. 1. All participants were administered Vitamin D orally in the included 18 RCTs based on their previous diet, physical activity, and medicines. The control groups received conventional therapy as before. The primary and demographic information of the eligible studies are presented in Tables 1, 2.

**Table 1:** Characteristics of included Randomized Controlled Trials.

Author	Year	Country	Participants (Male/Female)	Age(y) (Mean ± SD)	BMI kg/m^2^ (Mean ± SD)	Baseline Vitamin D Level (Mean ± SD)	Duration	Dose & Frequency	HOMA-IR (Mean ± SD)	Fasting Insulin (Mean ± SD)	Fasting Blood Glucose (Mean ± SD)
Anyanwu, A. C^15^	2016	Nigeria	T:17 C:16	T: 52.5 ± 2.2 C: 51.1 ± 1.9	NR	T: 6.9 ± 0.9 C: 6.4 ± 7.3 ng/mL	12 weeks	3000 IU/day	NR	NR	T: − 18.5 ± 43.7 C: 4.1 ± 63.4 mg/dL
Bazia r, N^16^	2014	Iran	T:41(28/13) C:40(26/14)	T: 50.34 ± 6.71 C: 52.75 ± 6.34	T: 27.33 ± 1.64 C: 27.25 ± 1.35	T: 14.33 ± 5.85 C: 15.50 ± 5.55 ng/mL	8 weeks	50,000 IU/week	T: −1.00 ± 2.05 C: 0.42 ± 1.69	T: − 2.09 ± 5.30 C: 0.3 3 ± 4.31 mU/L	T: − 15.05 ± 42.05 C: 9.06 ± 33.57 mg/dL
Calvo-Romero, J. M^17^	2016	Spain	T:28 C:28	71.7 ± 9.6	NR	10.6 ± 3.6 ng/mL	8 weeks	16,000 IU/ week	T: 1.24 ± 0.73 C: 1.42 ± 1.05	T: 8.93 ± 5.55 C: 9.97 ± 7.32 mU/L	T: 131.7 ± 30.4 C: 145.6 ± 35.5 mg/dL
Cojic, M^18^	2021	Montenegro	T:49(36/13) C:65(21/44)	T: 60.41 ± 8.5 C: 63.65 ± 8.2	T: 30.1 ± 4.6 C: 29.8 ± 5.0	T: 48.79 ± 31.63 C: 58.02 ± 32.32 nmol/L	24 weeks	50,000 IU / week × 3 months 14 000 IU / week × 3 months	T: − 0.23 ± 2.75 C: − 0.25 ± 3.3	T: 0.01 ± 7.05 C: 1.26 ± 8.39 mU/L	NR
Eftekhari, M. H^19^	2011	Iran	T:35 C:35	T: 53.8 ± 8.9 C: 52.4 ± 7.8	T: 28.3 4.4 C: 27.0 3.4	T: 38.5 ± 29.9n C: 43.3 ± 32.1 g/mL	12 weeks	20 IU/day	T: 1.22 ± 2.6 C: 1.41 ± 2.1	NR	NR
Gulseth, H. L^20^	2017	Norway	T:33(18/15) C:29(19/10)	T: 55.5 ± 9.2 C: 55.9 ± 9.2	T: 32.5 ± 5.1 C: 31.1 ± 4.7	T:38.0 ± 11.9 C:36.8 ± 12.6 nmol/L	24 weeks	400,000 IU/ week	NR	T: 1 ± 8 C: 21 ± 90 mmol/L	T: 0.6 ± 2.6 C: 0.6 ± 2.8 mmol/L
Imanparast, F^21^	2020	Iran	T:23(13/10) C:23(11/12)	T: 53.63 ± 12.29 C: 51.72 ± 9.11	T: 28.29 ± 2.64 C: 28.38 ± 2.14	T: 17.58 ± 8.86 C: 27.82 ± 19 ng/mL	16 weeks	50,000 IU/ week	T: − 0.61 ± 0.67 C: − 4.25 ± 2.44	T: − 0.63 ± 1.5 C: − 6.57 ± 6.62 IU/mL	T: − 13 ± 20.95 C: − 6 ± 15.19 mg/dL
Jorde, R^22^	2009	Norway	T:16(9/7) C:16(9/7)	T: 57.7 ± 9.7 C: 54.8 ± 5.9	T: 32.8 ± 6.8 C: 31.3 ± 6.3	T: 60.0 ± 14.0 C: 58.5 ± 21.0 nmol/L	24 weeks	40,000 IU/week	T: 0.3 ± 23.5 C: − 0.2 ± 13.7	T: –6 ± 251 C: –6 ± 116 pmol/L	T: − 0.2 ± 3.1 C: 0.4 ± 1.0 mmol/L
Kampmann, U^23^	2014	Denmark	T:7(6/1) C:8(2/6)	T: 61.6 ± 4.4 C: 57 ± 4.5	T: 35.3 ± 2.9 C: 32.4 ± 2.0	T: 31.0 ± 4.9 C: 34.8 ± 3.8 nmol/L	12 weeks	11,200 IU/day × 2 weeks, then 5600 IU/day × 10 weeks	NR	T: 12.6 ± 6.2 C: − 80.0 ± 75.4 pmol/L	T: 0.11 ± 0.4 C: 0.23 ± 0.6 pg/mL
Kim, H. J J^24^	2014	Korea	T:11 C:13	T: 73.27 ± 2.06 C: 70.08 ± 1.37	T: 24.08 ± 0.73 C: 23.72 ± 0.68	T: 10.44 ± 1.80 C: 11.66 ± 2.80 ng/mL	12 weeks	1200 IU/day	T: − 0.12 ± 0.36 C: 0.46 ± 0.37	T: − 0.43 ± 1.04 C: 1.37 ± 0.92 μU/mL	T: − 1.27 ± 10.83 C: 5.23 ± 9.39 mg/dL
Krul-Poel, Y. H^25^	2015	the Netherlands	T:129 (88/41) C:132(82/50)	T: 67 ± 8 C: 67 ± 9	T: 28.7 ± 4.6 C: 28.5 ± 4.5	T: 60.6 ± 23.3 C: 59.1 ± 23.2 nmol/L	24 weeks	50,000 IU/ month	NR	T: 0.7 ± 10.5 C: − 0.3 ± 9.9 mU/L	T: 0.4 ± 1.3 C: 0.2 ± 1.2 mmol/L
Mohammadi, S. M^26^	2016	Iran	T:32 C:32	T: 38.5 ± 6.8 C: 41.4 ± 6.9	NR	T: 19.0 ± 2.2 C: 23.0 ± 1.8 ng/ml	12 weeks	50,000 IU/ week	T: − 0.8 ± 0.6 C: 0.3 ± 0.25	T: − 3.5 ± 2.9 C: 0.23 ± 1.95 μU/mL	T: − 4.2 ± 14 C: − 2.7 ± 11.9 mg/dL
Nada, A. M^27^	2017	Egypt	T:41 C:41	52.7 ± 10.3	33.0 ± 6.1	14.0 ± 4.0 ng/mL	8 weeks	45,000 IU/ week	T: 2.6 ± 1.1 C: 4.7 ± 3.5	T: 8.5 ± 4.1 C: 12.9 ± 7.6 uU/mL	T: 7.9 ± 2.4 C: 9.1 ± 4.3 mmol/L
Safarpour, P^28^	2020	Iran	T:43(8/35) C:43(8/35)	T: 50.36 ± 10.2 C: 50.05 ± 10.7	T: 30.43 ± 3.23 C: 31.37 ± 3.40	T: 17.24 ± 7.83 C: 17.56 ± 7.82 ng/ml	8 weeks	50,000 IU/ week	T: 1.08 ± 3.48 C: 1.56 ± 3.58	T: 2.67 ± 6.57 C: 4.68 ± 7.73 micIU/mL	T: 3.04 ± 63.8 C: 7.56 ± 66.55 mg/dL
Tabesh, M^29^	2014	Iran	T:29 (15/14) C:30(14/16)	T: 50.2 ± 6.6 C: 51.0 ± 6.1	T: 30.5 ± 5.3 C: 30.3 ± 3.8	T: 28.0 ± 13.9 C: 45.7 ± 16.4 nmol/L	8 weeks	50,000 U/week	T: − 0.02 ± 0.17 C: 0.003 ± 0.17	NR	NR
Tamadon, M. R^30^	2018	Iran	T:30(19/11) C:30(19/11)	T: 60.1 ± 10.4 C: 65.1 ± 10.1	T: 28.4 ± 4.5 C: 25.8 ± 3.7	T: 15.2 ± 6.7 C: 15.4 ± 6.9 ng/mL	12 weeks	50,000 IU/ 2 week	T: –1.2 ± 1.8 C: 0.9 ± 2.3	T: − 3.4 ± 3.7 C: 2.0 ± 4.2 μIU/mL	T: − 13.8 ± 51.0 C: 6.7 ± 54.2 mg/dL
Witham, M. D^31^	2010	UK	T:19(12/7) C:21(16/5)	T: 65.3 ± 11.1 C: 66.7 ± 9.7	T: 33.3 ± 7.1 C: 31.1 ± 6.7	T: 41 ± 14 C: 45 ± 17 nmol/L	16 weeks	100,000 IU once	T: 4.2 ± 13.6 C: 4.2 ± 16.7	NR	NR
Yousefi Rad, E^32^	2014	Iran	T:28(15/13) C:30(21/9)	T: 50.03 C: 49.90	T: 27.94 ± 0.92 C: 28.75 ± 0.95	NR	8 weeks	4000 IU/day	T: − 0.14 ± 0.14 C: 0.22 ± 0.13	NR	NR

*NR* not reported, *T* treatment group, *C* control group, *UK* United Kingdom.

**Table 2:** Characteristics of included observational studies.

Author	Year	Country	Participants	Age(y) (Mean ± SD)	Baseline Vitamin D Level (Mean ± SD)	Correlation coefficient between VD and FI	Correlation coefficient between VD and HOMA-IR	Correlation coefficient between VD and FBG
Al-Daghri, N. M^33^	2013	KSA	153	50.2 ± 10.1	23.8 ± 1.5 nmol/L	− 0.28	− 0.23	− 0.25
Alharazy, S^34^	2021	Saudi Arabia	173	59.6 ± 6.8	14.2 ± 9.2 ng/mL	− 0.184	− 0.23	− 0.165
Cai, X^35^	2014	China	1408	57.1 ± 13.54	35.72 ± 13.64 nmol/L	NP	1.009	NP
Calvo-Romero, J. M^36^	2015	Spain	77	72.3 ± 9.8	NP	− 0.82	− 0.51	NP
Cătoi, A. F^37^	2021	Romania	47	33–68	NP	NP	− 0.02	NP
Choi, D. H^38^	2018	Korea	302	NP	15.1 ± 9.7 ng/mL	0.235	− 0.264	0.087
Dalgård, C^39^	2011	Denmark	668	72.5	NP	− 0.1	− 0.1	− 0.01
Dhas, Y^40^	2019	India	90	41.83 ± 5.91	16.68 ± 7.51 ng/mL	− 0.015	− 0.148	− 0.324
Fondjo, L. A^41^	2017	Ghana	118	58.81 ± 0.90	1.93–8.96 ng/mL	NP	− 0.153	NP
Gao, Y^42^	2015	China	395	59.62 ± 8.15	NP	− 0.045	NP	NP
Haidari, F. PhD^43^	2016	Iran	84	51.79 ± 9.01	NP	0.051	NP	− 0.21
Han, B^44^	2017	China	6597	52.5 ± 13.5	41.1 ± 10.8 nmol/L	NP	− 0.03	NP
Omar, D. F^45^	2018	Egypt	20	56.10 ± 1.39	NP	NP	− 0.301	− 0.549
Said, J^46^	2021	Kenya	128	56.2 ± 9.2	1.6–21.1 ng/mL	NP	0.07	NP
Tran Huu, T. T^47^	2021	Vietnam	110	69.86 ± 12.54	30.67 ± 8.55 mmol/L	NP	− 0.192	NP
Wang, W^48^	2019	China	106	50.12 ± 13.05	NP	NP	− 0.75	NP
Wang, W^49^	2018	China	264	50.04 ± 9.43	NP	− 0.039	− 0.042	− 0.002
Yang, Y^50^	2016	China	97	52.5 ± 10	36 ± 19 mmol/L	NP	− 0.22	NP
Zhang, J^51^	2021	China	109	49.79 ± 13.53	21.10 ± 10.39 ng/mL	NP	− 0.364	NP
Zhang, J^52^	2016	China	117	50.38 ± 13.47	21.40 ± 10.68 ng/mL	NP	− 0.327	NP

*NR* not reported, *KSA* Kingdom of Saudi Arabia, *FBG* Fasting Blood Glucose, *HOMA-IR* Homeostasis Model Assessment-Insulin Resistance, *FI* Fasting Insulin, *VD* Vitamin D.

### Quantitative data analysis

Results of the current meta-analysis proved that Vitamin D has shown significant effect on regulates insulin resistance, and there is a significant inverse association between serum levels of Vitamin D and insulin resistance (all P < 0.05). The comprehensive results are shown in Fig. 3.

#### Outcome of vitamin D supplementation

A total of 18 articles were included in the meta-analysis of the Vitamin D supplementation effect on IR. In the overall analysis, the diabetic with Vitamin D supplement treatment group showed significantly improve insulin resistance. As the results presented serum insulin (SMD = − 0.265, 95% CI − 0.394 to − 0.136, P < 0.05), glucose (SMD = − 0.17, 95% CI − 0.301to − 0.039, P < 0.05) and HOMA-IR (SMD = − 0.441, 95% CI − 0.582 to − 0.3, P < 0.05) with Vitamin D supplement compared with the routine treatment group, demonstrating that insulin resistance was alleviated. These results point not only to Vitamin D supplements can effectively relieve insulin resistance but also diabetes. However, there are significant heterogeneity between trials in the analysis results of insulin resistance (I^2^ = 86.7%, P < 0.05) and insulin (I^2^ = 91.3%, P < 0.05).

#### Correlation analysis results

In total, 20 articles were included in the meta-analysis to appraise the correlation of Vitamin D with insulin, glucose and HOMA-IR in T2DM patients. Those results showed that all three outcomes were significantly correlated in a negative manner with raised Vitamin D (insulin: r =  − 0.08 95% =  − 0.12 to − 0.04; glucose: r =  − 0.06 95% =  − 0.11 to − 0.01; HOMA-IR: r =  − 0.08 95% =  − 0.09 to − 0.06). A significant association was found between hypovitaminosis D and IR markers of type 2 diabetes development. It suggests that increasing serum Vitamin D levels can alleviate and inhibit the development of IR to a certain extent. Significant heterogeneity was observed in the correlation between Vitamin D and the three mainly markers from the Cochran’s Q test (insulin: I^2^ = 93.7%, P < 0.05; glucose: I^2^ = 77.0%, P < 0.05; HOMA-IR: 89.5%, P < 0.05). This means the relationship of vitamin status and markers of T2DM, HOMA-IR in particular, are not independent of other variables (Figs. 3). Further subgroup analysis and sensitivity analysis are required.

### Subgroup and sensitivity analyses

Subgroup analyses and sensitivity analyses were performed to ascertain the primary origin of heterogeneity by changing the chosen parameters simultaneously. Pre-planned subgroup analyses for some parameters were performed for the primary outcomes with a representation number of eligible trials. A pairwise meta-analysis of the results by subgroup is provided in the Table 3 for the primary outcomes (Supplementary Fig. 1). Due to the diversity of interventions, subgroup analysis is required for duration and dosages of the therapies. Moreover, the potential effect of Vitamin D deficiency appears to be prominent among persons at risk for diabetes. Insufficient data on the effect of confounding factors (such as assay methods, nationalities, human resources and ethnicities) prevented us from pursuing other preplanned subgroup analyses. Then, we performed subgroup analysis according to different dosages, duration, and Vitamin D levels. We separated these activity events into two categories according to different doses, matching the high-dose group (≥ 50,000 IU/week) and the low-dose group (< 50,000 IU/week), respectively. The duration was divided into two subgroups: short-term subgroup (< 12 weeks) and long-term subgroup (≥ 12 weeks). As per the Endocrine Society guidance, vitamin D deficiency, insufficiency, and replete were defined as 25(OH)D levels of < 50 (< 20 ng/mL), 50–75 (21–29 ng/mL), and > 75 nmol/L (≥ 30 ng/mL), respectively^53^. More comprehensive analysis on subgroups with a representative number of trials yielded some intriguing results. The results of subgroup analyses for the effect of vitamin D supplementation on IR and the relationship between serum levels of vitamin D and IR in T2DM have been summarized in Table 3, and the detailed results are described below.

**Table 3:** Summary of subgroup analysis with random effects SMD (95% CI).

Subgroups		FBG	FI	HOMA-IR	Correlation coefficient between VD and HOMA-IR
Dose	< 50,000 IU/week	− 3.9(− 0.63, − 0.16)	− 0.70(− 0.96, − 0.440	− 0.66(− 0.89, − 0.43)	NA
	≥ 50,000 IU/week	− 0.7(− 0.23, 0.09)	− 0.12(− 0.27, 0.03)	− 0.32(− 0.49, − 0.14)	NA
Duration	< 4 months	− 0.34(− 0.52, − 0.15)	− 0.67(− 0.86, − 0.47)	− 0.81(− 0.99, − 0.64)	NA
	≥ 4 months	0.01(− 0.18, 0.19)	0.07(− 0.1, 0.25)	0.20(− 0.03, 0.43)	NA
Vitamin D levels	Replete	NA	NA	− 0.08(− 0.55, 0.39)	NA
	Insufficiency	0.11(− 0.12, 0.34)	0.09(− 0.14, 0.32)	0.03(− 0.67, 0.72)	− 0.36(− 0.49, − 0.04)
	Deficiency	− 0.31(− 0.47, − 0.15)	− 0.43(− 0.58, − 0.27)	− 0.5(− 0.65, − 0.35)	− 0.05(− 0.07, − 0.03)

*NA* not analyzed, *FBG* Fasting Blood Glucose, *HOMA-IR* Homeostasis Model Assessment-Insulin Resistance, *FI* Fasting Insulin, *VD* Vitamin D.

*Statistically significant variables at P value < 0.05.

Subgroup analysis results for the two primary outcomes of blood glucose and insulin shown that the low-dose, short-term and Vitamin D deficiency subgroups were statistically significant compared with other subgroups (all P < 0.05). In terms of HOMA-IR subgroup analysis results, the statistically significant treatment results were observed within each of the subgroups (all P < 0.05), except for the long-term, replete, and insufficiency subgroup (P ≥ 0.05). Due to the limited number of observational studies, only subgroup analysis of HOMA-IR was performed. There is a negative correlation between HOMA-IR and Vitamin D in both deficient and insufficiency groups. From the overall and subgroup analysis, the results showed that adding Vitamin D supplementation in the conventional treatment group had a certain effect on diabetes, and vitamin D deficiency has been associated with increased risk of T2DM. Based on the above analysis, we speculate that low-dose Vitamin D supplementation (< 50,000 IU/week) in patients with T2DM and hypovitaminosis D may be more effective. After subgroup analysis, we discovered that heterogeneity was remained considerably high when compared to previous studies. We therefore performed further Sensitivity Analyses for each end point by excluding individual studies. The results of the sensitivity-pooled SMD on the bulk of the outcomes indicated that all exclusions had no effect on the prior analyses results.

### Publication bias and quality assessment

Review authors’ judgments about each risk-of-bias item for all the eligible studies using the Cochrane Collaboration tool^54^ to assess the methodological quality and bias.

**Figure 2:**
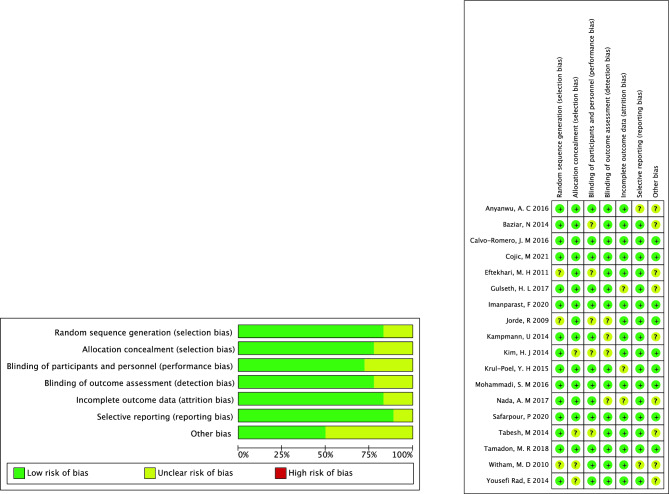
Overall summary of risk of bias in the included studies. + : Low risk of bias; − : High risk of bias; ?: Unclear risk of bias.

All 18 RCT studies included in the review were assessed for bias risk. The results of the risk of bias assessment shown that 6 studies had a low risk of bias (high-quality), 12 studies had a moderate risk of bias (moderate-quality), and none had a high risk of bias (low-quality). The detailed results of each item are presented in Fig. 2. The funnel plots for of Vitamin D supplementation outcomes and correlation analysis results were generally symmetrical, suggesting insignificant publication bias (Supplementary Fig. 2). Egger’s test for HOMA-IR (P = 0.406) and fasting insulin (P = 0.551) suggested insignificant publication bias in RCTs studies. Publication bias for HOMA-IR was evaluated insignificant (Egger’ test: P = 0.292). The Egger’s graphical test suggested that there was significant publication bias for other indicators. The overall major conclusions did not alter following the elimination of studies having a high risk of bias in the cumulative analysis.

## Discussion

**Figure 3:**
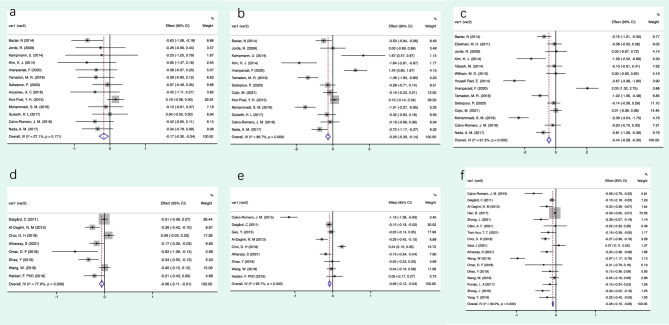
Mean difference in the changes in Glycolipid metabolism indexes. (**a**) FBG; (**b**) FI; (**c**) HOMA-IR; (**d**) Correlation coefficient between VD and FBG; (**e**) Correlation coefficient between VD and FI; (**f**) Correlation coefficient between VD and HOMA-IR. *FBG* Fasting Blood Glucose, *HOMA-IR* Homeostasis Model Assessment-Insulin Resistance, *FI* Fasting Insulin, *VD* Vitamin D.

Given IR linked to multitude of disorders, the potential effectiveness and optimal concentration of vitamin D consumption on risk IR and subsequent T2DM has attracted the interest of many research fields. From the overall results, it is evident from current meta-analysis that there are significant inverse correlations (all P < 0.05, Fig. 3) between the intervention and placebo groups showing vitamin D supplementation may improve HOMA-IR, FI and FBG levels, which may suggest biologically significant trends favoring vitamin D supplementation. We therefore speculate that Vitamin D deficiency belongs to a key factor accelerating the development of IR and the risk of hyperglycemia as well. There were no published studies specifically designed to assess the safety and effectiveness of long-term and mega-dose vitamin D administration to reduce risk factors of T2DM; therefore, no firm conclusions can be drawn regarding the effectiveness of vitamin D on T2DM^55^. As logic would predict, information on serum vitamin D level accounted for differences in IR better than intake of vitamin D. The assessment of serum vitamin D level is less subject to measurement error than is the assessment of dietary and supplemental vitamin D intake alone. Thus, blood concentrations rather than the assessment of dietary intake provide the most accurate assessment of individual vitamin D status. The potential of reverse causation is a primary limitation of observational studies; causality hence cannot be established. Therefore, evidences from long-term randomized controlled trial would be needed to determine the issue of causality and to rigorously evaluate the effectiveness of vitamin D. We thus conducted a meta-analysis included RCTs and observational studies with the expectation of more precisely defining the relationship alternations observed in either analysis alone.

### Serum VD levels and IR

The serum 25(OH)D is a sensitive biomarker for the vitamin D status and is the major circulating form of vitamin D, reflecting the amount of vitamin D obtained from both dietary sources and cutaneous synthesis. Studies have shown that vitamin D has direct and/or indirect effects on multiple mechanisms related to the pathophysiology of T2DM. Based on pre-clinical and animal studies, vitamin D supplementation seems to been proved improving insulin resistance and playing a regulatory role in insulin secretion and beta-cell survival^56–58^, while VD deficiency seems to impair glucose-stimulated insulin secretion and pancreatic beta cells function^59–61^. An additional evidence indicated vitamin D also regulates extracellular calcium concentration and flux through the pancreatic beta cell^62^, and the function of calbindin, cytosolic calcium-binding protein as modulator of insulin release in pancreatic beta cells^63,64^. Since insulin secretion is a calcium dependent pathway^65^, persistent alterations in calcium flux could affect the insulin secretory response^66,67^. A tremendous amount correlational studies have found that serum level of vitamin D was significant inversely correlated with IR^68–70^, indicating that subjects with higher vitamin D had higher IR index and which is similar to the results of this study. Other cross-sectional studies shown conflicting results with failing to find a significant relationship^71,72^. Furthermore, few meta-analyses had found vitamin D was effective in decreasing risk for diabetes^73^, and hypovitaminosis D is related to increased levels of insulin resistance^74^. Clearly, correlational researches have produced mixed results. The substantial differences findings could be mainly because surveys had differed significantly in their designs, population characteristics, inclusion criteria, sampling frames, and recruitment strategies have included a variety of techniques. Additionally, some correlational studies did not adjust for potential confounders, which might have affected the overall results. More prospective multicenter and large-scale clinical trials are required to verify the results.

### Supplemental VD interventions and IR

There are increasing, largely inconsistent literatures had investigated the effect of dietary and supplementary vitamin D in association with IR/IS, glycemic indices, and similar metabolic outcomes^75–77^. In the current clinical trials, vitamin D supplementation has attracted significant attention for the following reasons: (1) administer easily; (2) controlled precisely; (3) effect significantly^76,77^. Therefore, consumption of vitamin D via supplements must be administered to raise serum concentrations significantly. Since the prevalence of vitamin *D* deficiency is very frequent among patients with diabetes, whereas the effect of vitamin D supplementation on its development history is largely unknown. The results of the current meta-analysis showed that IR was significantly relieved in those who did take supplemental vitamin D compared with those who not (Fig. 3).

However, the most clinical cross-sectional studies investigating the impact of vitamin D supplementation on the control of glucose homeostasis, the risk of prediabetes, and prevalence and severity of T2D complications had yielded inconsistent results^78^. Even most research has suggested that vitamin D supplementation could not improve IR. In recent three large trials designed and conducted specifically to evaluate the prevention of diabetes in patients with vitamin D supplementation, when compared with placebo, estimated a 10% to 13% reduction in fall risk developing diabetes in persons with prediabetes without vitamin D deficiency^79–81^. And few meta-analyses had showed Vitamin D supplementation was shown to reduce insulin resistance effectively^82^. Meanwhile, Lee CJ et al. have found a modest reduction of HbA1C and no difference in FBG after vitamin D treatment in T2MD^83^. A systematic review with fifteen trials had reported there was no effect of vitamin D supplementation on glycemic or insulin resistance outcomes^84^. However, estimating this variation is difficult due to differences in dosage, dosage form, duration, and populations, as well as a most of potential epigenetic confounders. Above all, the vitamin D supplementation is safe, low-cost and easy-to-comply-with^85^, it has the potential to represent a novel strategy for T2DM prevention and treatment. The meta-analysis results could play a certain role in help patients with T2DM tailored dietary patterns and direct healthcare organizations in building fast-acting preventative and treatment plans for those who are at risk for prediabetes.

### Risk of developing insulin resistance

Many studies have paid attention to the influence of Vitamin D on preventing DNA hypermethylation, subsequent functional inactivation of genes and other epigenetic alterations in pancreatic beta cells and other insulin sensitive peripheral tissues/ organs. The incidence of IR has been considered as the main pathogeny of T2DM increased in vitamin D deficiency and decreased after exogenous supplementation of vitamin D^86^. In studies of prediabetes individuals, found that supplementing with vitamin D reduced progression to diabetes and increased reversal from normoglycemia in subjects with prediabetes^87,88^. Moreover, observational studies have shown that serum vitamin D status seem to be inversely and significantly correlated with most other IR disorders and the risk of T2DM reported to date^89,90^. It is reasonable to hypothesize Vitamin D deficiency is a key factor in accelerating the onset and development of IR and consequently T2DM as well. A linear trend analysis result has shown that a 4 ng/ml increment in Vitamin D levels was associated with a 4%(95% CI 3 to 6; P < 0.05) lower risk of T2DM^90^. The medical research council Ely prospective study has reported that baseline serum vitamin D levels were inversely related to 10-year risk of hyperglycemia, which could be predictive of future glycemic status and IR^91^.

Moreover, clinical studies have shown that vitamin D linked to surrogate parameters of cardiovascular damage^92^ and mediated reduction in albuminuria^93^, suggesting the possible role of vitamin D in slowing progression of diabetic nephropathy and cardiovascular diseases. Based those evidences presented above it is reasonable to hypothesize that vitamin D could exert vascular protective activities to against onset and development of micro- and macrovascular complications in T2DM patients^94^. Since vitamin D deficiency is widespread and heavily impacts worldwide^95^, and is be correlated with the incidence of T2DM and its complications, it is recommended to determine the vitamin D levels of T2DM patients. Besides, Vitamin D has been implicated in development and progression of various diseases^96^, including cancer, immunologic diseases, cardiovascular diseases, and other chronic diseases. Prevention and correction of vitamin D deficiency is highly desired. Further studies are required gain more detailed data needed for the full clinical utilization of vitamin D supplementation as a promising adjuvant therapy for T2DM patients.

### Strengths and weaknesses

Research articles were mined through a systematic search, which is one of the meta-analysis' major strengths. Grading of Recommendations Assessment, Development, and Evaluation (GRADE) was used to evaluate observational studies according to the gold standard international methodology (Supplementary Table 3). However, we cannot rule out the possibility of potentially confounding factors, which are the main limitations. Potentially confounding factors include the patient characteristics (such as age, ethnicity, lifestyle) and the significant variation in plasma vitamin D levels of the participants. As all RCTs trials, intervention studies have treated subjects with variability in dosage, dosage forms, durations. The drawback in all observational studies is that the exposures were not blind and random. Other residual confounding factors could not be excluded either, such sun exposure, which have not been mentioned in all studies. Based on the mentioned above, those confusions may be the main source of this study limitations. We judged the evidence to be moderate on the basis of the current review strengths and weaknesses.

## Conclusion

The pathophysiologic, preventive and therapeutic effects of Vitamin D have been proposed and interpreted. In this meta-analysis and systematic review, we strive to organize and clarify current information about any potentially eligible studies in this field, revealing several important findings. Results proved that Vitamin D has shown significant effect on regulates insulin resistance, and there is a significant inverse association between serum levels of Vitamin D and insulin resistance. Furthermore, the biological plausibility behind the potential association and the effect of vitamin D supplementation on IR were described. Thus, serum vitamin D levels could be most predictive of IR, and individuals with low vitamin D concentrations may be helped by increasing vitamin D levels, typically by diet and supplements. Vitamin D supplementation is expected to be integrated into conventional medical approaches as a promising adjuvant therapy for T2DM patients and to mitigate the burden of diabetes for individuals and society.

## Supplementary Information

Supplementary Figure 1.

Supplementary Figure 2.

Supplementary Table 1.

Supplementary Table 2.

Supplementary Table 3.

## Data Availability

No new data were created or analyzed in this study. Data sharing is not applicable to this article. QZ should be contacted if someone wants to request the data from this study. On request, data were extracted from original research and data used in meta-analyses are accessible.
