# Supplementary material for: Serum and supplemental vitamin D levels and insulin resistance in T2DM populations: a meta-analysis and systematic review
Source: Sci Rep. 2023 Jul 31;13:12343. doi: 10.1038/s41598-023-39469-9 (PMC10390579; doi:10.1038/s41598-023-39469-9)
Supplement: Supplementary file 1 — Supplementary Figure 1. [file 41598_2023_39469_MOESM1_ESM.docx]

Supplementary Figure 1. Summary of subgroup analysis with random effects SMD (95% CI). ^*^Statistically significant variables at P value < 0.05. (A) dose subgroup of Fasting Blood Glucose, (B) duration subgroup of Fasting Blood Glucose, (C) vitamin D levels subgroup of Fasting Blood Glucose, (D) dose subgroup of Fasting Insulin, (E) duration subgroup of Fasting Insulin, (F) vitamin D levels subgroup of Fasting Insulin, (G) dose subgroup of HOMA-IR, (H) duration subgroup of HOMA-IR, (I) vitamin D levels subgroup of HOMA-IR, (J) vitamin D levels subgroup of Correlation coefficient between VD and HOMA-IR.

C

B

A

F

E

D

H

I

G

J
