# Supplementary material for: Serum and supplemental vitamin D levels and insulin resistance in T2DM populations: a meta-analysis and systematic review
Source: Sci Rep. 2023 Jul 31;13:12343. doi: 10.1038/s41598-023-39469-9 (PMC10390579; doi:10.1038/s41598-023-39469-9)
Supplement: Supplementary file 2 — Supplementary Figure 2. [file 41598_2023_39469_MOESM2_ESM.docx]

Supplementary figure 2. Funnel plot for publication bias analysis of the selected studies.

B

A

C

E

D

F

(A) FBG; (B) FI; (C) HOMA-IR; (D) Correlation coefficient between VD and FBG; (E) Correlation coefficient between VD and FI; (F) Correlation coefficient between VD and HOMA-IR. Abbreviations: FBG: Fasting Blood Glucose; HOMA-IR: Homeostasis Model Assessment-Insulin Resistance; FI: Fasting Insulin; VD: Vitamin D.
