# Supplementary material for: Serum and supplemental vitamin D levels and insulin resistance in T2DM populations: a meta-analysis and systematic review
Source: Sci Rep. 2023 Jul 31;13:12343. doi: 10.1038/s41598-023-39469-9 (PMC10390579; doi:10.1038/s41598-023-39469-9)
Supplement: Supplementary file 4 — Supplementary Table 2. [file 41598_2023_39469_MOESM4_ESM.docx]

Supplementary table 2. search strategy

Search: **((Vitamin D) AND (diabetes)) AND (insulin resistance)**

("vitamin d"[Supplementary Concept] OR "vitamin d"[All Fields] OR "ergocalciferols"[Supplementary Concept] OR "ergocalciferols"[All Fields] OR "vitamin d"[MeSH Terms] OR "ergocalciferols"[MeSH Terms]) AND ("diabete"[All Fields] OR "diabetes mellitus"[MeSH Terms] OR ("diabetes"[All Fields] AND "mellitus"[All Fields]) OR "diabetes mellitus"[All Fields] OR "diabetes"[All Fields] OR "diabetes insipidus"[MeSH Terms] OR ("diabetes"[All Fields] AND "insipidus"[All Fields]) OR "diabetes insipidus"[All Fields] OR "diabetic"[All Fields] OR "diabetics"[All Fields] OR "diabets"[All Fields]) AND ("insulin resistance"[MeSH Terms] OR ("insulin"[All Fields] AND "resistance"[All Fields]) OR "insulin resistance"[All Fields])

**Translations**

**Vitamin D:** "vitamin d"[Supplementary Concept] OR "vitamin d"[All Fields] OR "ergocalciferols"[Supplementary Concept] OR "ergocalciferols"[All Fields] OR "vitamin d"[MeSH Terms] OR "ergocalciferols"[MeSH Terms]

**diabetes:** "diabete"[All Fields] OR "diabetes mellitus"[MeSH Terms] OR ("diabetes"[All Fields] AND "mellitus"[All Fields]) OR "diabetes mellitus"[All Fields] OR "diabetes"[All Fields] OR "diabetes insipidus"[MeSH Terms] OR ("diabetes"[All Fields] AND "insipidus"[All Fields]) OR "diabetes insipidus"[All Fields] OR "diabetic"[All Fields] OR "diabetics"[All Fields] OR "diabets"[All Fields]

**insulin resistance:** "insulin resistance"[MeSH Terms] OR ("insulin"[All Fields] AND "resistance"[All Fields]) OR "insulin resistance"[All Fields]
