# Supplementary material for: Serum and supplemental vitamin D levels and insulin resistance in T2DM populations: a meta-analysis and systematic review
Source: Sci Rep. 2023 Jul 31;13:12343. doi: 10.1038/s41598-023-39469-9 (PMC10390579; doi:10.1038/s41598-023-39469-9)
Supplement: Supplementary file 5 — Supplementary Table 3. [file 41598_2023_39469_MOESM5_ESM.docx]

Supplementary Table **3**. Grading of Recommendations Assessment, Development, and Evaluation (GRADE) approach evidence certainty and summary of findings of the clinical important outcomes.
